# Impact of DNA damaging agents on genome-wide transcriptional profiles in two marine *Synechococcus* species

**DOI:** 10.3389/fmicb.2013.00232

**Published:** 2013-08-16

**Authors:** Sasha G. Tetu, Daniel A. Johnson, Deepa Varkey, Katherine Phillippy, Rhona K. Stuart, Chris L. Dupont, Karl A. Hassan, Brian Palenik, Ian T. Paulsen

**Affiliations:** ^1^Department of Chemistry and Biomolecular Sciences, Macquarie UniversitySydney, NSW, Australia; ^2^Microbial and Environmental Genomics Department, J. Craig Venter InstituteRockville, MD, USA; ^3^Marine Biology Research Division, Scripps Institution of Oceanography, University of CaliforniaSan Diego, CA, USA; ^4^Microbial and Environmental Genomics Department, J. Craig Venter InstituteSan Diego, CA, USA

**Keywords:** cyanobacteria, *Synechococcus*, transcriptome, microarray, toxic stress, ethidium bromide, mitomycin C, DNA damage

## Abstract

Marine microorganisms, particularly those residing in coastal areas, may come in contact with any number of chemicals of environmental or xenobiotic origin. The sensitivity and response of marine cyanobacteria to such chemicals is, at present, poorly understood. We have looked at the transcriptional response of well characterized *Synechococcus* open ocean (WH8102) and coastal (CC9311) isolates to two DNA damaging agents, mitomycin C and ethidium bromide, using whole-genome expression microarrays. The coastal strain showed differential regulation of a larger proportion of its genome following “shock” treatment with each agent. Many of the orthologous genes in these strains, including those encoding sensor kinases, showed different transcriptional responses, with the CC9311 genes more likely to show significant changes in both treatments. While the overall response of each strain was considerably different, there were distinct transcriptional responses common to both strains observed for each DNA damaging agent, linked to the mode of action of each chemical. In both CC9311 and WH8102 there was evidence of SOS response induction under mitomycin C treatment, with genes *recA*, *lexA* and *umuC* significantly upregulated in this experiment but not under ethidium bromide treatment. Conversely, ethidium bromide treatment tended to result in upregulation of the DNA-directed RNA polymerase genes, not observed following mitomycin C treatment. Interestingly, a large number of genes residing on putative genomic island regions of each genome also showed significant upregulation under one or both chemical treatments.

## Introduction

Ocean environments comprise a vast component of the Earth's biosphere and play a key role in global biogeochemical processes. Unicellular marine cyanobacteria, including *Synechococcus* and *Prochlorococcus* species, are estimated to constitute 20–40% of total marine chlorophyll biomass and carbon fixation, and hence significantly impact the carbon cycle and global climate processes (Partensky et al., 1999). Considering their ecological importance, it is important to understand what stresses these organisms are susceptible to and how they respond to these challenges.

In recent years work has begun to unravel the gene level responses of *Synechococcus* and *Prochlorococcus* to a range of environmental stressors. Nitrogen limitation is thought to be common in the marine environment and these microbes have regulatory systems such as NtcA for regulating their responses to nitrogen (Lindell and Post, 2001). Phosphate limitation stress is also an issue in some marine environments and a subset of cyanobacteria encode the PhoB/R two component regulatory systems for responding to this (Tetu et al., 2009). Another challenge is high irradiance, especially UV wavelengths (Llabres and Agusti, 2006), and adaptation to high light conditions in marine *Synechococcus* involves expression of genes whose products are involved with dissipation of excess light, scavenging to eliminate reactive oxygen species and changes to photosynthetic machinery (Scanlan et al., 2009; Mella-Flores et al., 2012). Marine cyanobacteria have been shown to be more sensitive to copper stress than eukaryotic phytoplankton and their response to this stress has been characterized at the transcriptional level (Stuart et al., 2009). Iron, which is necessary for photosynthetic apparatus, is also considered to be important in the ecology of marine cyanobacteria, and transcriptional responses to changing iron availability have been characterized in *Prochlorococcus* strains (Thompson et al., 2011).

In contrast, relatively few studies to date have focused on how marine cyanobacteria respond to toxic chemicals, despite the finding of multidrug-like efflux systems in marine cyanobacterial genomes (Palenik et al., 2003). Numerous chemicals, including both xenobiotics and compounds produced by other marine microbes potentially impact these important primary producers, particularly in coastal regions. Toxicity tests on marine *Synechococcus* have indicated that these species are particularly sensitive to exposure to the herbicide atrazine, possibly due to their small cell size and relatively high surface area to volume ratio (Weiner et al., 2004).

Genome sequences are now available for a number of marine cyanobacteria, including *Synechococcus* sp. WH8102, isolated from tropical Atlantic Ocean waters (Palenik et al., 2003), *Synechococcus* sp. CC9311 isolated from coastal waters (Palenik et al., 2006) as well as a number of others from habitats including the Sargasso Sea, Red Sea and the Mediterranean (Palenik et al., 2006; Dufresne et al., 2008). The availability of whole genome sequences allows comparative genomic analyses and techniques such as microarray analysis to be employed to elucidate mechanisms by which cyanobacteria cope with different challenges, such as exposure to toxic compounds.

To gain insight into how different strains of *Synechococcus* respond to toxic chemical exposure, microarray experiments were carried out on an open ocean and a coastal strain using ethidium bromide (EB) and mitomycin C (MC). For each chemical a “shock” treatment (2 h exposure) was applied, as examining short time courses reduces secondary effects due to long term toxicity. These two compounds were chosen as they are both DNA damaging agents which have different modes of action that have been well characterized previously. EB binds to DNA by intercalation between base pairs in the DNA helix, resulting in inhibition of DNA-directed RNA synthesis (Richardson, 1973). MC is a potent DNA cross-linker known to be capable of inducing the SOS response system in many bacterial species (Janion, 2008) and has been used to induce cyanobacterial prophages in environmental samples [for example (Sode et al., 1994)]. The SOS response, which occurs after single stranded DNA accumulates in a cell, is a global response to DNA damage that results in induction of DNA repair and mutagenesis pathways. In well studied bacteria, such as *Escherichia coli*, it is known to result in activation of RecA, which in turn inactivates the LexA repressor, decreasing the pool of this protein which during normal growth negatively regulates a set of genes including *umuD* and *umuC* involved in mutagenic repair (Janion, 2008). While some cyanobacteria such as the well studied freshwater species *Synechocystis* sp. PCC 6803 have lost the classic *E. coli* type LexA mediated SOS response (Domain et al., 2004), recent analyses of marine cyanobacteria *Prochlorococcus marinus* PCC9511 and *Synechococcus* sp. WH7803 have demonstrated a typical, coordinated SOS response to DNA damage, involving *lexA* and *recA* (Kolowrat et al., 2010; Blot et al., 2011).

Whilst neither EB nor MC is currently considered as a significant environmental pollutant and to our knowledge there is at present no information regarding levels of either chemical in the environment, both have been synthesized for more than 50 years, inevitably resulting in some degree of environmental exposure. Mitomycin C has a long history of use as an antitumor drug in the treatment of a wide range of cancers and is marketed in most countries worldwide (Bradner, 2001). In addition to a long history of routine laboratory use as a DNA stain, EB has been widely used in veterinary medicine to treat cattle infected with trypanosomes, a use which continues in parts of the world despite recent concerns regarding its mutagenicity (Roy Chowdhury et al., 2010). EB has also been widely used as a marker for multidrug efflux [for example (Mitchell et al., 1999; Patel et al., 2010; Hassan et al., 2011)] which makes it a particularly good test compound to gain comparative insights into the role of putative efflux pumps encoded within *Synechococcus* genomes in detoxification of the cell.

We hypothesized that a coastal *Synechococcus* strain would have a more robust response to chemical toxicant stress since xenobiotic compounds [likely sources include stormwater, industrial and agricultural runoff (Nogales et al., 2011)] and other toxic chemicals [for example antibiotics produced by marine bacteria (Long et al., 2003)] are likely to be in higher concentrations in coastal environments.

## Methods

### Strains and growth conditions

**Figure A1:**
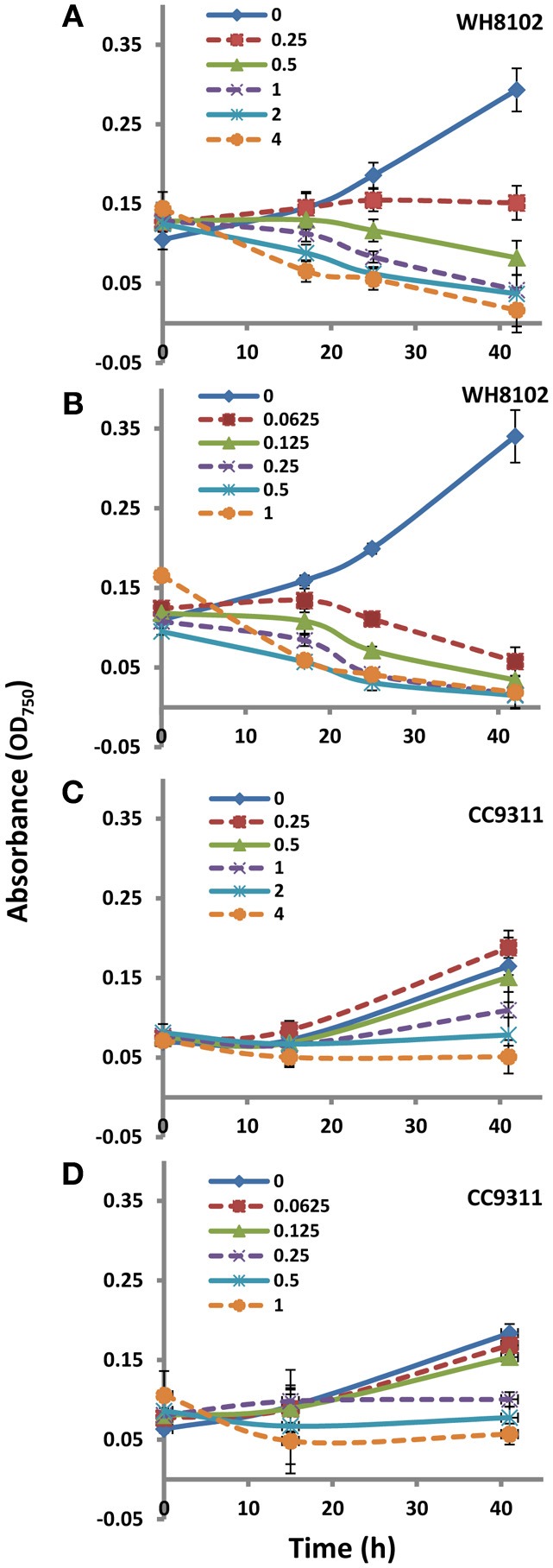
**Growth of WH8102 and CC9311 on treatment with EB and MC at concentrations ranging from 0–4 and 0–1 μg/ml^−1^ respectively. (A)** Growth of WH8102 on treatment with EB; **(B)** Growth of WH8102 on treatment with MC; **(C)** Growth of CC9311 on treatment with EB; **(D)** Growth of CC9311 on treatment with MC. The axes represent absorbance at 750 nm against time in hours. The experiment was conducted in triplicate and the mean plotted. The error bars represent standard deviation.

Axenic *Synechococcus* sp. WH8102 and *Synechococcus* sp. CC9311 cultures (referred to as WH8102 and CC9311) were maintained in either SN (Waterbury and Willey, 1988) medium made with seawater from the Scripps Pier (Scripps Institution of Oceanography, La Jolla, CA), or in an artificial seawater medium (SOW) prepared as described previously (Su et al., 2006) with 9.0 mM NaNO_3_ standard. For growth assays, batch cultures were grown in glass flasks, gently stirred, or in glass tubes, at 25°C under 30 μmol photons m^−2^ s^−1^ continuous white light. Chemical treatments were performed using each compound at a concentration that was inhibitory to long-term growth, 2 μ g/mL for EB and 0.5 μ g/mL for MC (Figure A1). To evaluate cell health at the time which chemical treated cells were harvested, flow cytometry (FACSCalibur, Becton Dickinson) was used to examine cell size and pigmentation. To investigate how these strains respond to these chemical treatments in terms of photosynthetic performance, a saturation pulse (200 ms) was applied to cells using a PHYTO-PAM fluorescence measuring system equipped with an emitter-detector-cuvette assembly unit ED-101US/D (Walz) to determine the quantum yield of photosystem II at multiple time points after the addition of each compound.

### RNA isolation

*Synechococcus* strains were grown in 1.5-liter batch cultures to the early exponential phase (approximately 1.5 × 10^8^ cells/ml), and half the culture was centrifuged at room temperature at 10,400 × g, and immediately resuspended in Trizol reagent (Invitrogen), then frozen at −80°C for RNA extraction in parallel with treated cells. EB (final concentration 2 μg/mL) or MC (final concentration 0.5 μ g/mL) were added to the remaining culture. After a two hour incubation, these cells were harvested and suspended in Trizol reagent as outlined above. Total RNA was extracted from the cell pellet using Trizol reagent (Invitrogen) according to the manufacturer's instructions. The RNA was resuspended in 100 μ l of DEPC-treated water. Using the RNeasy Mini kit (Qiagen) and following the manufacturer's protocol, the RNA was twice digested with DNase I (Qiagen) then eluted from the columns. The purity and yield of RNA was determined spectrophotometrically by measuring optical density at wavelengths of 260 and 280 nm. Samples were stored at −80°C.

### DNA microarray transcriptional profiling

Full genome microarrays were synthesized for both WH8102 and CC9311 as described previously (Tetu et al., 2009). For WH8102 this consisted of a mixed population of PCR amplicons (2142 genes) and 70-mer oligonucleotides (389 genes), while for CC9311 arrays were constructed with 70-mer oligonucleotides for each of 2,892 genes. Each gene was represented six times on an array. Negative controls were 50% DMSO–50% deionized water, and positive controls included Arabidopsis PCR amplicons and 70-mer oligonucleotides. Each microarray experiment included a minimum of two biological replicates and a minimum of three technical replicates for each biological sample, and at least one “dye-swap” experiment per biological replicate.

An indirect labeling method was used to label cDNA as previously described (Peterson et al., 2004), where cDNA is synthesized in the presence of a nucleoside triphosphate analog containing a reactive aminoallyl group to which a fluorescent dye molecule, either cyanine 3 or cyanine 5 (Cy3/Cy5) is coupled. Approximately 4 μg of total RNA was used for indirect labeling, leading to the production of approximately 4 μg of cDNA with approximately 200 pmol of dye molecule incorporated per microgram of cDNA synthesized. Prior to hybridization, labeled cDNA was scanned spectrophotometrically to ensure optimal dye incorporation per sample for adequate signal intensity.

All hybridizations were performed as previously described (Peterson et al., 2004). Processing of the TIFF images from hybridized arrays was performed using TIGR-Spotfinder (www.tigr.org/software), and the datasets normalized by applying the LOWESS algorithm, using block mode and a smooth parameter of 0.33, available in the TIGR-MIDAS package (www.tigr.org/software). Statistical analysis was performed on the mean of log_2_-transformed signal ratios of the replicate spots using the Statistical Analysis of Microarrays (SAM) algorithms (Tusher et al., 2001) with a false discovery rate of less than 1%. Subsequent analyses considered genes significantly up- or downregulated based on a cutoff of 0.4-log_2_-fold/ −0.4-log_2_-fold change. Descriptions of the microarray experiments, quantitation data, and array design have been deposited into the gene expression omnibus (GEO) database (http://www.ncbi.nlm.nih.gov/geo/) and have been assigned accession number GSE39818. Genome map circular figures were generated to show the location of the up- and downregulated genes in each *Synechococcus* genome using CGview software (Stothard and Wishart, 2005).

To identify sets of genes which displayed similar patterns of expression when exposed to different test conditions, *k*-means clustering analysis (Soukas et al., 2000) was performed using the TIGR Multi-Experiment Viewer TMEV software (Saeed et al., 2003). Gene trees were subsequently generated by average linkage hierarchical clustering with Euclidean distance as the distance metric.

### Cloning and expressing sync2766+7

CC9311 gene sync_2766 (putative RND multidrug efflux transporter) was PCR amplified independently and together with adjacent sync_2767 (putative MFP subunit) using forward primers sync_2766F (5′-CAC CAT GGA ATG CCA GTC AAA ATT CTC-3′) and sync_2767F (5′-CAC CAT GAT TTT GCG GCT TCA ATC G -3′) respectively with reverse primer sync_2766R (5′-GTT TTC AGA AGT ATC TGG CAA AGA GTG-3′). PCR products were generated from CC9311 genomic DNA using Phusion polymerase with HF buffer using the following cycling parameters: 30 cycles (98°C, 10 s; 58°C, 30 s; 72°C, 2 min 20 s) after an initial denaturation at 98°C for 30 s. Products were cloned into Invitrogen Gateway pENTR/SD/D-TOPO vector and shuttled into pET-DEST42 following manufacturers' instructions [and as described previously (Ding et al., 2005)]. Plasmids containing cloned genes were sequenced prior to analysis to confirm the absence of deleterious mutations.

Immunoblots were conducted on whole cell lysates of *E. coli* BL21 cells harboring the pET-DEST42 vector with sync_2766 alone and together with sync_2767 or a control pET-DEST42 vector containing *Pseudomonas aeruginosa* PA01 gene PA0220, a putative amino acid transporter (referred to as the negative control). Cells were grown to mid-exponential phase and gene expression induced with 0.1 mM isopropyl-β-d-thiogalactopyranoside (IPTG) for 1 h at 37°C. Samples were run on a 10% SDS-PAGE gel and transferred to a PVDF membrane. Heterologously expressed V5-tagged proteins were detected using anti-V5 monoclonal antibody conjugated with horseradish peroxidase (HRP) (1/5000 dilution). The membranes were developed with chromogenic substrate 4-Chloro-1-Naphthol (4CN).

Broth-dilution MIC analyses were used to evaluate the resistance potential of the putative RND transporter in *E. coli* BL21(DE3). These analyses were conducted in cells cultured in Mueller-Hinton broth, using standard methodology as described elsewhere (Wiegand et al., 2008). The concentration ranges tested for MC and EB were 0–5 and 0–200 μ g/mL respectively.

## Results and discussion

### Global transcriptional response to ethidium bromide and mitomycin C exposure

**Figure A2:**
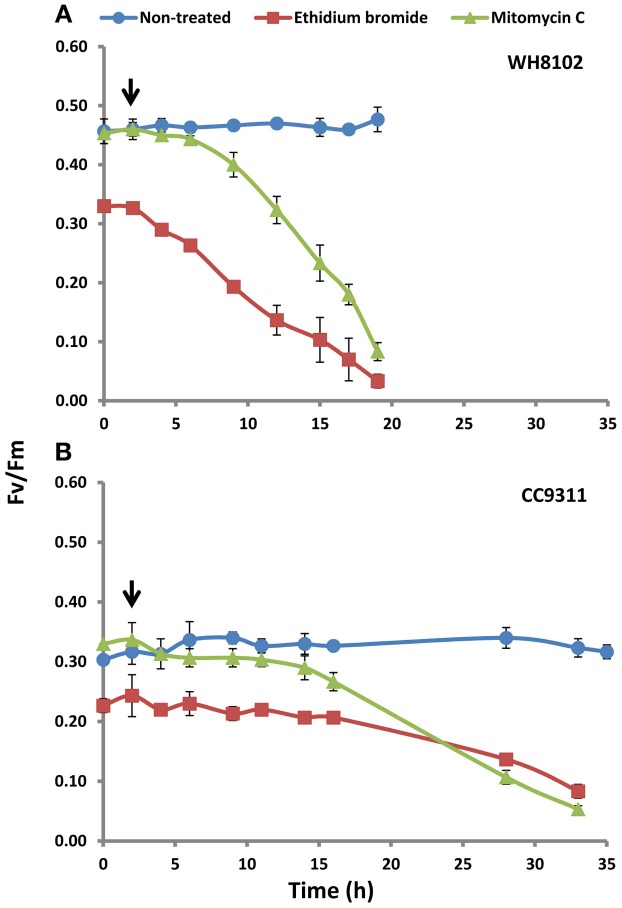
**Photosynthetic efficiency of photosystem II in (A) WH8102 and (B) CC9311 on treatment with EB and MC, and with no treatment as determined using PAM fluorometry.** The axes represent Fv/Fm (ratio of variable fluorescence and maximum fluorescence) against treatment time in hours. The experiment was carried out in triplicate and the mean plotted. The error bars represent standard deviation. The arrow represents the point of 2 h exposure at which RNA was isolated for microarray analysis.

A total of four sets of global expression microarray experiments were conducted examining the response of coastal strain CC9311 and open-ocean strain WH8102 to exposure to DNA damaging agents EB and MC. In both strains the mRNA levels of a large number of genes was strongly altered by each shock treatment, compared to the control (Table 1). *Synechococcus* CC9311 showed a particularly high degree of gene transcription up- and downregulation in response to the tested compounds, with more than 200 genes strongly (>2 fold) up- and downregulated by each treatment. (Lists of all significantly up- and downregulated genes for each strain and stress treatment are presented in Table S1). At the time point that gene expression was examined (2 h post addition) chemical treatments had not impacted average cell size or pigmentation as assessed by flow cytometry (data not shown). Measurements of photosynthetic quantum yield for each strain after chemical addition indicated that major reduction in photosynthetic activity occurred after the 2 h time point at which RNA was harvested (Figure A2).

**Table 1:** **Numbers of genes in *Synechococcus* sp. WH8102 and CC9311 whose transcription was strongly (more than twofold) upregulated or downregulated under EB and MC shock treatments as detected by microarray analysis**.

**Strain**	**EB**		**MC**	
	**Upregulated**	**Downregulated**	**Upregulated**	**Downregulated**
WH8102	97 (317)	77 (382)	93 (236)	80 (301)
CC9311	255 (786)	228 (781)	211 (715)	226 (745)

*Total numbers of significantly affected genes are given in brackets*.

### Transcriptional responses varied for genes in different functional categories

To explore each organism's response to the applied toxic chemical stresses, genes showing significant changes in transcription levels were grouped according to clusters of orthologous groups (COG) functional categories (Table 2). As expected, genes in the “DNA replication, recombination and repair” grouping tended to be transcriptionally upregulated in both strains under both stress conditions. In CC9311 genes in the “posttranslational modification, protein turnover, chaperone” category also tended to be transcriptionally upregulated, whilst in WH8102 these genes were disproportionately downregulated, particularly under conditions of EB stress. For example, both strains have genes encoding molecular chaperone DnaK2 heat shock protein homologs (SYNW2508 and sync_2923). In CC9311 this gene was significantly upregulated under both stress conditions, while the homolog in WH8102 was significantly downregulated in the EB treatment (no significant change in MC treatment). Genes encoding proteases FtsH2 and FtsH3 (sync_0355 and sync_0825/ SYNW0305 and SYNW1587) DnaJ protein (sync_0023 and SYNW0024) and co-chaperone GrpE (sync_0022 and SYNW0023) similarly showed transcriptional upregulation in CC9311 and downregulation or no significant change in WH8102.

**Table 2:** **Proportion of *Synechococcus* sp. CC9311 and WH8102 genes assigned to each COG category that were significantly transcriptionally upregulated and downregulated by EB or MC treatment**.

	**COG function**	**CC9311**				**WH8102**			
		**EB**		**MC**		**EB**		**MC**	
		**% ↑**	**% ↓**	**% ↑**	**% ↓**	**% ↑**	**% ↓**	**% ↑**	**% ↓**
Q	Secondary metabolites biosynthesis, transport and catabolism	36	21	14	36	17	0	13	17
P	Inorganic ion transport and metabolism	23	25	32	26	16	27	10	18
I	Lipid metabolism	34	34	18	28	7	13	9	13
H	Coenzyme metabolism	23	28	20	28	3	16	5	7
F	Nucleotide transport and metabolism	18	42	18	25	0	15	0	15
E	Amino acid transport and metabolism	21	32	23	25	9	25	3	14
G	Carbohydrate transport and metabolism	13	30	19	31	7	20	9	14
O	Posttranslational modification, protein turnover, chaperones	37	18	32	17	10	32	10	9
C	Energy production and conversion	18	32	20	34	10	34	6	20
U	Intracellular trafficking and secretion	25	19	13	6	12	24	12	0
N	Cell motility and secretion	8	15	31	31	13	0	0	13
M	Cell wall/membrane/envelope biogenesis	27	20	27	18	10	14	9	7
T	Signal transduction mechanisms	25	40	22	36	17	14	10	17
D	Cell division and chromosome partitioning	16	47	16	47	14	5	0	0
L	DNA replication, recombination and repair	36	20	34	13	15	7	15	1
K	Transcription	33	25	21	25	15	13	6	13
J	Translation, ribosomal structure and biogenesis	24	21	16	37	9	17	5	19
V	Defense mechanisms	41	34	31	25	10	10	13	3
R	General function prediction only	27	21	31	15	10	10	8	6
S	Function unknown	26	32	26	23	9	12	4	8
	Not in COG	28	27	25	27	18	11	13	14

Interestingly, in CC9311 transcription of “secondary metabolite biosynthesis, transport and catabolism” related genes was affected differently by the two toxins, showing a tendency toward upregulation in EB and downregulation in MC treatment (Table 2). A high proportion of genes in the “energy production and metabolism” grouping were transcriptionally downregulated in both strains, although the trend was especially pronounced in WH8102. The “nucleotide transport and metabolism” category genes were also observed to be disproportionately downregulated in both treatments in both strains (Table 2).

### DNA damage agents activated the accessory genomes of both CC9311 and WH8102

**Figure 1:**
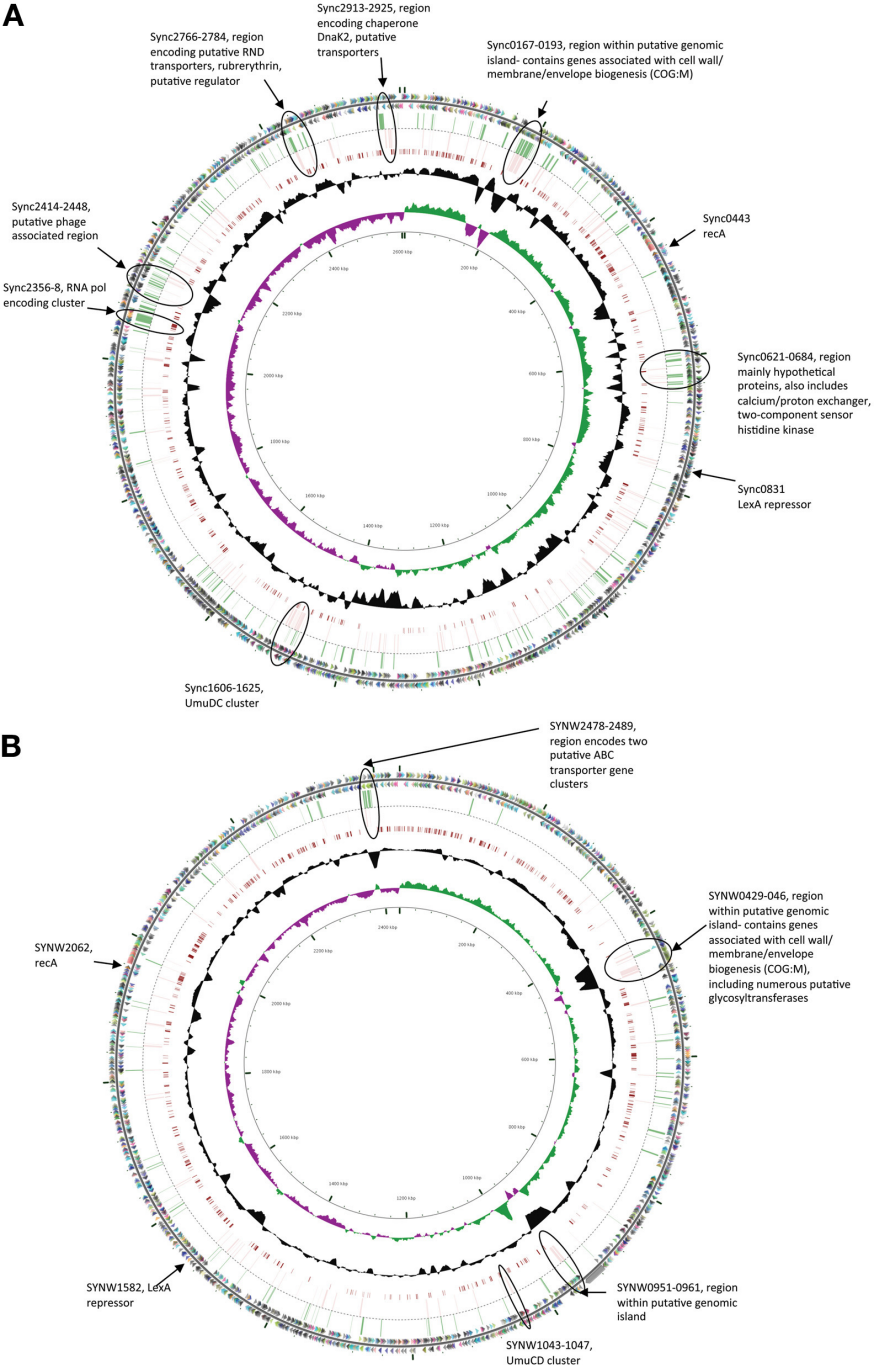
**Circular figures showing chromosomes of *Synechococcus* sp. CC9311 (A) and WH8102 (B) indicating location of genes strongly upregulated (>2 fold) by EB and MC treatments.** Outermost two circles indicate positions of CDSs in plus (circle 1) and minus (circle 2) strands colored by functional category: translation, ribosomal structure, and biogenesis (pink); transcription (orange); DNA replication, recombination and repair (fuchsia); cell division and chromosome partitioning (yellow); posttranslational modification, protein turnover, chaperones (brown); cell wall/membrane/envelope biogenesis (olive); cell motility and secretion (dark green); inorganic ion transport and metabolism (green); signal transduction mechanisms (blue/green); energy production and conversion (aqua); carbohydrate transport and metabolism (blue); amino acid transport and metabolism (teal); nucleotide transport and metabolism (turquoise); coenzyme metabolism (dark blue); lipid metabolism (lavender); secondary-metabolite biosynthesis, transport, and catabolism (purple); general function prediction only (light gray); function unknown (dark gray); and no COG (gray black). Genes whose expression was upregulated by more than 2 fold in microarray experiments when exposed to EB are indicated by green bars in the third circle, while those similarly upregulated by MC are indicted by pink bars in the fourth circle. Moving toward the center, the next circle map pairwise blastn alignments (expected threshold = 1e-20) between CC9311 and WH8102. Circle six shows G + C content (deviation from average), and the seventh circle illustrates G + C skew in green (+) and purple (−). The scale (in Kbp) is indicated on the innermost circle. Genes of particular interest are circled and described.

In both strains many genes within regions previously flagged as putative genomic islands (Palenik et al., 2003, 2006) showed significant transcriptional upregulation in either one or both stress treatments (Figure 1). Fourteen genome regions in WH8102 have been identified with anomalous G + C content, ten of which were adjacent to putative phage integrases, potentially representing phage associated genomic islands (Palenik et al., 2003). While these regions contain less than 10% of the ORFs in this genome, these putative islands contained a surprisingly large proportion of the highly upregulated genes. In the case of EB 37% of genes upregulated by more than 2 fold (36 of 97 genes) were located in such regions, while for MC stress the proportion was greater still with 47% of such genes located in putative genomic islands (44 of 93 genes upregulated by more than 2 fold). In WH8102 three of the non-phage associated island regions are of particular interest. The region which spans SYNW0424 to SYNW0460 contains a large number of genes which are highly upregulated under MC stress (Figure 1B). Many of the affected genes are predicted to function in carbohydrate modification of the cell envelope (COG:M), including numerous genes annotated as putative glycosyltransferases. The gene region SYNW0951 to SYNW0961 also contains a majority of genes strongly upregulated by MC stress [one exception being SYNW0953 encoding a giant 1.2MDa motility-related protein (Strom et al., 2012)] and to a lesser extent EB stress (most show significant upregulation, but falling below the 2 fold cutoff), however, there is almost no functional information for this set of genes. A third island region spanning SYNW2477 to SYNW2491 contains a cluster of genes strongly upregulated by EB (Figure 1B). This cluster spans the two previously mentioned putative ABC transporter operons predicted to be involved in transport of zinc and cyanate.

In CC9311 previous genome analyses flagged 19 regions of atypical trinucleotide composition as potential genomic islands (Palenik et al., 2006). Again these regions contained a higher than average proportion of the highly upregulated genes in both stress treatments. While these genomic islands contain roughly 4% of the encoded putative ORFs, more than 10% of genes upregulated by >2 fold in each of the stress treatments were within these genomic regions (28 of 255 in the EB treatment and 26 of 211 in MC treatment). Again, certain island regions were observed to contain clusters of highly upregulated genes. Most notably the large genomic island region spanning sync_0167 to sync_0193, includes a large number of genes showing high levels of upregulation in one or both stress treatments (Figure 1A). Similar to the large island in WH8102, this island contains a large proportion of genes associated with modification of the cell envelope (COG:M), including genes involved with capsular polysaccharide biosynthesis. Another putative recently horizontally acquired region showing a concentration of highly upregulated genes is the phage associated gene cluster sync_2414 to sync_2448, which encodes mainly genes of unknown function (Figure 1A). Putative RND multidrug efflux transporter encoding genes sync_2766 and sync_2767, which were upregulated by more than 2 fold in both stress treatment, are also located in a putative genomic island region.

Interestingly, other stress treatments in cyanobacterial strains have also resulted in a disproportionally high impact on transcription of genes within genomic island regions. In *Prochlorococcus* MED4 and MIT9313 a large fraction of the differentially expressed genes in response to iron starvation were observed to reside on genomic islands and/or hypervariable regions (Thompson et al., 2011). Copper shock experiments in *Synechococcus* CC9311 also resulted in upregulation of a large number of genes in genomic island regions (Stuart et al., 2009), and recent examination of particular genes in these island regions has shown genomic island genes can confer adaptive advantage to stress conditions (Stuart et al., 2013).

### Comparison of DNA damage stress responses for CC9311 and WH8102

Many of the orthologous genes shared by these two *Synechococcus* genomes showed different transcriptional responses in the two test strains following each shock treatment. In CC9311 many of the genes observed to be strongly downregulated (>2 fold) in EB stress have orthologs in WH8102 (155/288), however, only 10 of these orthologs were strongly downregulated in WH8102 (Table A1). Similarly for the 112 strongly upregulated CC9311 genes which have orthologs, only eight of these also showed greater than 2 fold upregulation in WH8102. The results of the MC shock treatment were similarly divergent between the two strains (Table A1). While a smaller number of WH8102 genes with orthologs in CC9311 showed high (>2 fold) up and downregulation, again only a small proportion of the CC9311 orthologs responded in the same way (Table A1). In all experiments a small number of orthologous genes responded in the opposite fashion in the two tested strains, for example showing strong upregulation in one strain and strong downregulation in the other, or vice versa (Table A1). In previous work looking at the response of these two *Synechococcus* strains to copper shock there was also only a modest overlap in significantly regulated orthologous genes (Stuart et al., 2009).

Previous genome analyses of both strains have shown a distinct difference in the two-component regulatory systems of these strains; WH8102 encodes relatively few histidine kinase sensors, only five compared to CC9311, which has 11 (Palenik et al., 2006). Analysis of the transcriptional response of these sensor kinase genes revealed significant upregulation of six of these genes in CC9311 in at least one of the toxicant exposure conditions, while none of the WH8102 genes were significantly upregulated in either condition (Table 3). Sensor kinase sync_0675 in CC9311 showed particularly high levels of upregulation on exposure to both MC and EB, to our knowledge there has been no previous work into the conditions regulating this gene. These observations suggest that the coastal strain CC9311 has different regulatory circuits to WH8102, which may allow it to react to conditions by switching on or off transcription of a greater proportion of “core” *Synechococcus* genes, potentially contributing to the higher tolerance/resilience of CC9311 to the tested toxic compounds.

**Table 3:** **Transcriptional response of sensor kinases to toxic shock treatments in *Synechococcus* sp. CC9311 and WH8102**.

**CC9311 sensor kinase**	**MC fold change (log_2_)**	**EB fold change (log_2_)**	**WH8102 homolog**	**MC fold change (log_2_)**	**EB fold change (log_2_)**
sync_0675	1.32 (±0.26)	1.4 (±0.37)			
sync_1079	0.36 (±0.22)	0.7 (±0.43)			
sync_0573	−1.32 (±0.25)	−0.95 (±0.40)			
sync_2219	0.46 (±0.19)	NS	SYNW0551	NS	−0.59 (±0.16)
sync_1006	−0.7 (±0.18)	−0.9 (±0.65)	SYNW0753	NS	−0.35 (±0.18)
sync_0706	−0.25 (±0.09)	NS			
sync_1133	NS	0.81 (±0.43)			
sync_0668	0.61 (±0.16)	NS			
sync_0263	−0.5 (±0.23)	−0.92 (±0.44)			
sync_0286	NS	1.37 (±0.40)	SYNW0246	NS	NS
sync_1233	NS	NS	SYNW0807	NS	NS
			SYNW0948^*^	−0.15 (±0.06)	NS

* *SYNW0948 is involved in phosphate sensing and has no CC9311 homolog*.

### Transcriptional responses are linked to the “mode of action” of chemical toxicants

In both strains a set of genes showing a significant transcriptional response to both DNA damaging agents was observed. In CC9311 there were 88 genes strongly (more than 2 fold) upregulated and 107 strongly downregulated by both tested compounds. While many of these genes are presently annotated as hypothetical proteins, of those with functional assignations, there were a number encoding regulatory and transport related proteins. These include the two-component response regulator (sync_0115), transcriptional regulator MarR family (sync_2782), two type II alternative RNA polymerase sigma factors (sync_0098, sync_1018) and the sensor histidine kinase (sync_0675) previously discussed. In addition, the genes encoding the protease FtsH2 (sync_0355) and components of a putative RND multidrug efflux transporter (sync_2766 and sync_2767) were transcriptionally upregulated. The strong transcriptional increase observed for the regulatory protein-encoding genes in CC9311 may account for the large proportion of this genome which is affected by these stresses, as these regulators may affect transcription of numerous other genes. WH8102 encodes a homolog only for sync_0115, gene SYNW0126, which itself was not significantly upregulated. In WH8102 there were 22 genes strongly upregulated and 23 strongly downregulated by both tested compounds, almost all of which have not been ascribed a function. Those few with functional predictions include an encoded ribosomal protein SYNW2091, ABC transporter component SYNW2479 and CpeT homolog SYNW2003.

While there were overlaps in the transcriptional response between the two shock treatments, there were also clear differences in the response to each chemical, many of which can be accounted for by differences in the mode of action of these compounds. MC is a potent DNA crosslinker and is known to be capable of inducing the SOS response system in many bacterial species (Janion, 2008), while EB binds to DNA by intercalation between base pairs in the DNA helix, resulting in inhibition of DNA-directed RNA synthesis (Richardson, 1973). In both strains there was evidence of SOS response induction under MC treatment. In both CC9311 and WH8102 genes encoding RecA (sync0443/ SYNW2062), the LexA repressor (sync0831/ SYNW1582) and UmuC (sync1607/SYNW1043) were significantly upregulated under MC but not under EB treatment. Another gene, sync1474/SYNW1405, encoding a conserved hypothetical protein present in both examined strains was also highly upregulated under MC treatment but not in the EB experiment. This gene, whose function is presently unknown, appears to be specific to cyanobacteria and has homologs in most sequenced *Synechococcus* genomes. In CC9311 *umuD* encoding sync1606 was also significantly upregulated by MC stress (and to a lesser degree in the EB treatment), while the homolog in WH8102 (SYNW1044) was not. Interestingly, in CC9311 there is a cluster of genes downstream of *umuDC* that were also significantly upregulated in the MC treatment (sync1608-sync1613, sync1615, sync1620-sync1623) (Figure 1A). However, as most of these encode proteins of unknown function, it is again difficult to speculate on the exact role of this cluster, which may encode a clade specific SOS response.

It has previously been suggested that cyanobacteria lack an SOS response similar to that of *E. coli* (Domain et al., 2004; Patterson-Fortin et al., 2006) based largely on work in the model freshwater species *Synechocystis* sp. PCC6803, where LexA appears to instead be involved in carbon assimilation. In this strain the *lexA* gene is not DNA damage inducible and its amino acid sequence shows loss of residues required for activity of archetypal LexA proteins, which function to repress expression of DNA repair genes in other prokaryotes (Patterson-Fortin et al., 2006). However, recent experimental and bioinformatic analyses have indicated that it is likely that many of the small, marine cyanobacterial species do indeed have an inducible pathway involved in DNA repair, similar to the *E. coli* SOS response and involving LexA. Li and colleagues examined the available 33 sequenced cyanobacterial genomes and found that, while a small number, including *Synechocystis* sp. PCC6803, did not harbor a recognizable *lexA* gene, the majority do encode a LexA homolog with conserved DNA-binding domains (Li et al., 2010). In their analysis of UV stress response in *Prochlorococcus marinus* PCC9511, Kolowrat and colleagues found that classic, *E. coli*-like DNA repair pathways appeared to operate, noting conservation of active residues in LexA and the presence of a putative LexA binding site upstream of *recA*, *umuC*, *umuD* and *lexA*, and hypothesizing this strain possesses an active SOS response mechanism involving the *lexA* gene (Kolowrat et al., 2010). Similarly, *Synechococcus* sp. WH7803 was recently shown to respond to oxidative stress via up-regulation of a putative LexA regulon, which includes *lexA*, *recA* and *umuDC* (Blot et al., 2011).

In their work Li and colleagues computationally predict *lexA* regulons for CC9311 and WH8102 based on the location of putative LexA boxes within the operator region of a set of genes (Li et al., 2010). Interestingly, only a subset of these genes were found to be significantly upregulated under MC stress in this work, 15 out of 27 for CC9311 and 10 out of 46 for WH8102 (Table 4) indicating that further refinement of the LexA boxes using experimental information may be possible.

**Table 4:**
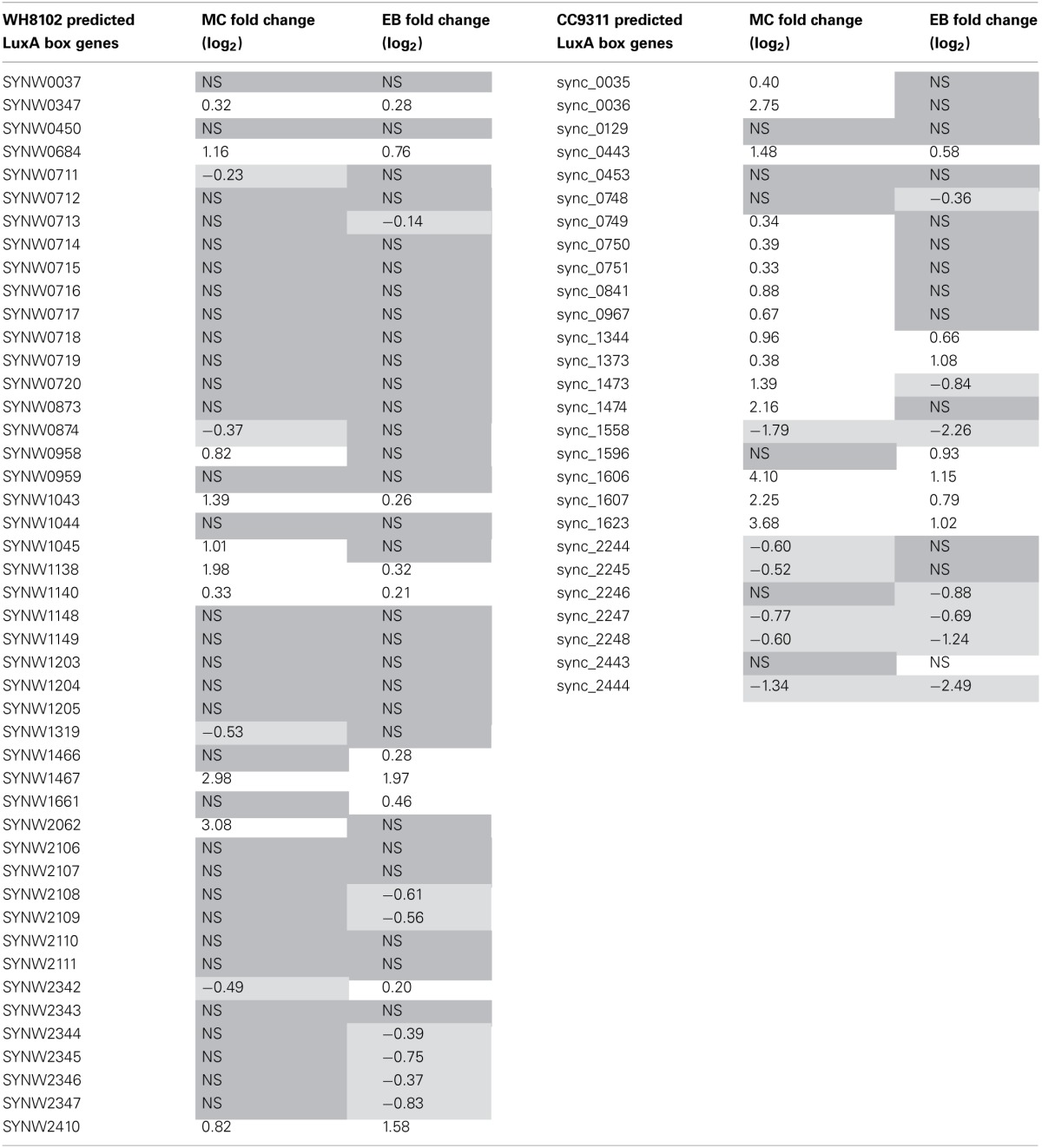
**Transcriptional response of *Synechococcus* sp. WH8102 and CC9311 genes previously flagged as having predicted LexA boxes (Li et al., 2010)**.

*Dark grey shading indicates instances where gene transcript levels were not significantly different between the control and test conditions. Light grey shading indicates gene transcript levels observed to be significantly reduced in the test condition compared to the control values*.

EB, which acts as an inhibitor of DNA-directed RNA synthesis, led to significant transcriptional upregulation of genes encoding DNA-directed RNA polymerases in both organisms. In CC9311 EB treatment resulted in strong upregulation of the DNA-directed RNA polymerase gene cluster sync_2356-2358 and a number of surrounding genes (Figure 1A), none of which were significantly upregulated by MC. In WH8102 two out of three genes in the homologous DNA-directed RNA polymerase gene cluster were significantly upregulated in the EB experiment (SYNW0613 and 0614 but not 0615), but to a lower degree, showing log_2_ fold changes 0.76 and 1.0, respectively. Part of this effect in both species is that EB may be generally inhibiting transcription and the cell is responding by additional DNA-directed RNA polymerase production. In WH8102 EB treatment also resulted in upregulation of two adjacent sets of genes encoding putative ABC transporter encoding operons SYNW2479-2481 and SYNW2485-2487, annotated as putative zinc and cyanate transporters respectively. All but one of these genes (SYNW2479) were not significantly upregulated in the MC treatment. Only two of these genes have homologs in CC9311 (sync_1497/SYNW2479 and sync1498/SYNW2480) and in CC9311 these genes were not significantly affected by EB.

### Does sync2766 encode a RND multidrug efflux transporter?

Among the set of CC9311 genes highly upregulated in both DNA damage treatments were sync_2766 and sync_2767 which are annotated as putative RND multidrug efflux transporter and membrane fusion protein (MFP) subunit respectively. Gene sync_2766 contains a conserved domain AcrB (COG0841), indicative of a cation/multidrug efflux pump, while sync_2767 contains the conserved domain RND_mfp (TIGR01730). These genes reside in a region of the genome which was previously flagged as a putative genomic island and protein BLAST searches with the translated amino acid sequences reveal sporadic distribution in marine cyanobacteria (data not shown). We performed a series of expression studies using sync_2766 cloned independently and together with sync_2767 into expression vector pET-DEST42 in *E. coli* BL21(DE3) cells to look for evidence of efflux pump activity. Expression was observed by immunoblotting only when both sync_2766 and sync_2767 were present in the construct. Minimum inhibitory concentration (MIC) assays were carried out with both EB and MC, however, growth of cells expressing sync_2766-7 was not significantly different to the negative control. For EB MICs of 15 μg/mL were routinely observed for both control and experimental constructs, while MICs of 0.09 μg/mL were recorded for MC. In gram-negative cells, high-level drug resistance mediated by RND efflux systems, typically relies on the formation of tripartite complexes that include an inner-membrane pump (such as sync_2766), a MFP (such as sync_2767) and an outer-membrane spanning channel, which may be distally encoded in the genome (Nikaido and Takatsuka, 2009). High-level resistance may not have been observed from Sync_2766/7 in *E. coli*, since this organism lacks a cognate outer-membrane protein for this transport system. Alternatively, it is possible that the high intrinsic resistance of *E. coli* to these compounds (relative to *Synechococcus* sp.), may have swamped any efflux activity attributable to sync_2766.

### *Synechococcus* Spp. display unique responses to different stress conditions

Comparing the results of this study to previous microarray experiments on the response of marine *Synechococcus* to nutrient limitation [phosphate starvation (Tetu et al., 2009), (Ostrowski et al., 2010)] heavy metal stress [copper toxicity (Stuart et al., 2009)] and nickel starvation (Dupont et al., 2012), it appears that the transcriptional response to different “types” of stress tend to be quite specific and can differ substantially between strains. In each study the set of genes showing the highest transcriptional increase differed. In many cases this is not surprising as the function of these genes was clearly linked to the nature of the induced stress, for example increasing transcription of alkaline phosphatases under phosphate starvation conditions and transcriptional activation of SOS response genes after MC treatment.

In the analysis of global transcriptional responses of *Synechococcus* strains CC9311 and WH8102 to copper toxicity (Stuart et al., 2009) it was shown that the coastal strain CC9311 responded with a more strain specific oxidative or copper acclimation response than the open-ocean strain WH8102, where a more generic stress response was observed. It was also noted that many of the copper toxicity responsive genes in CC9311 may have been acquired by horizontal gene transfer, however, these genes reside in different island regions to those observed to be responsive to EB and MC. Interestingly, many of the Cu-stress induced WH8102 genes flagged as generic stress response genes, including chaperone proteins DnaJ/DnaK, GroEL/GroES, HtpG and endopeptidase Clp, were observed in this work to be strongly downregulated under conditions of EB toxicity.

One common response observed for both strains following stress with high-copper concentrations and both DNA damaging agents applied in this work was the tendency for photosystem genes to be downregulated. Stuart et al. (2009) reported that of the 37 total photosystem genes, 14 were downregulated in CC9311 while 10 genes were downregulated in WH8102 under high-copper shock conditions, and none of these photosystem genes were upregulated in either strain under these conditions. In this study CC9311 was observed to downregulate 26 photosystem genes under conditions of EB toxicity and 28 when exposed to MC. The open ocean strain WH8102 downregulated 12 photosystem genes when exposed to EB and 24 when exposed to MC. In both cases, however, there were a small number of significantly upregulated photosystem genes (in CC9311 five genes following EB exposure and two genes following MC exposure, in WH8102 three genes following EB exposure). Measurements of photosystem II quantum yield, generated by pulse amplitude modulated fluorometry using the saturation pulse method, indicated that these transcriptional changes occurred before physiologically detectable changes in photosynthesis (Figure A2). Genes involved with nitrate transport also showed a tendency to downregulation in both DNA damage stress and copper shock treatments, with putative nitrate transporters SYNW2463-4 and sync_2899 strongly downregulated in both studies.

**Figure 2:**
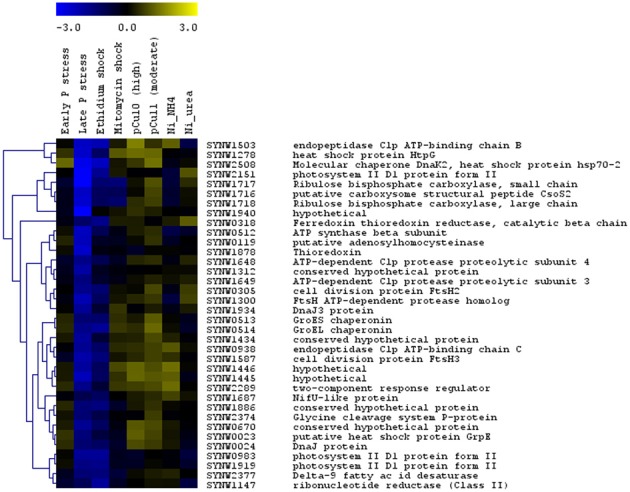
**Hierarchical trees showing clusters of *Synechococcus* sp. WH8102 genes whose expression was affected similarly by growth under a large number of stress conditions.** The expression levels for this strain under eight different stress conditions: early and late phosphate stress (Tetu et al., 2009), EB and MC shock (this study), moderate and high copper shock (pCu11; pCU10) (Stuart et al., 2009) and Nickel (Ni) deprivation in cultures growing on NH4^+^ and urea (Dupont et al., 2012) were compared. For each set of experiments data from two independent biological replicates was used. The color bar indicates the log_2_ ratio.

The large number of datasets concerning transcriptional responses of WH8102 to a range of stress conditions [copper toxicity (Stuart et al., 2009), phosphate starvation (Tetu et al., 2009) and nickel starvation (Dupont et al., 2012)] enabled us to conduct cluster analyses which revealed a cluster of stress related genes upregulated in most treatments (Figure 2). This set of upregulated genes encodes functions such as Clp endopeptidase, heat shock proteins, chaperones and cell division proteins. However, as noted above, these were typically downregulated in the EB treatment. This may be due to the possibility that EB directly inhibits transcription (interestingly, late phosphate stress similarly led to downregulation of these genes).

## Conclusions

*Synechococcus* sp. WH8102 and CC9311 are useful model marine cyanobacteria and can be used to look at how related organisms which live in different environments (open and coastal ocean, respectively) respond to changes such as exposure to toxicants, nutrient deprivation or other stresses. Genome sequences are available for both of these strains, which have provided insight into the genetic basis for adaptations to their different marine environments. Coastal aquatic environments tend to be more dynamic than open ocean environments and, fitting with this, coastal strain CC9311 was previously observed to have genetic mechanisms which may impart a greater capacity to sense and respond to changes in its environment than open ocean strain WH8102 (Palenik et al., 2006). In this work the two tested strains showed a number of differences in their transcriptional response to each of the DNA damage agents tested. Coastal strain CC9311 was observed to alter transcription of a much greater proportion of the genome in response to both toxic compounds tested. Examination of the orthologous genes shared between the strains showed that genes in CC9311 were more likely to illicit a transcriptional response than their WH8102 counterparts. The coastal strain may be able to elicit a more effective global transcriptional response to these compounds through upregulation of two-component response regulators, which could act to coordinate the transcriptional changes observed in this genome.

The data presented here extends our understanding of how model *Synechococcus* strains respond to different stress conditions at the global transcriptional level and helps validate SOS regulatory elements in these strains. Our results indicate that strains residing in different niches, e.g., coastal compared to open ocean environments, may elicit quite different global responses to different encountered stresses. Given the importance of these primary producers to global carbon cycling and marine ecosystem health it is important to continue to extend our understanding of *Synechococcus* stress responses, especially regarding potential anthropogenic pressures.

### Conflict of interest statement

The authors declare that the research was conducted in the absence of any commercial or financial relationships that could be construed as a potential conflict of interest.

## Figures and Tables

**Table A1:**
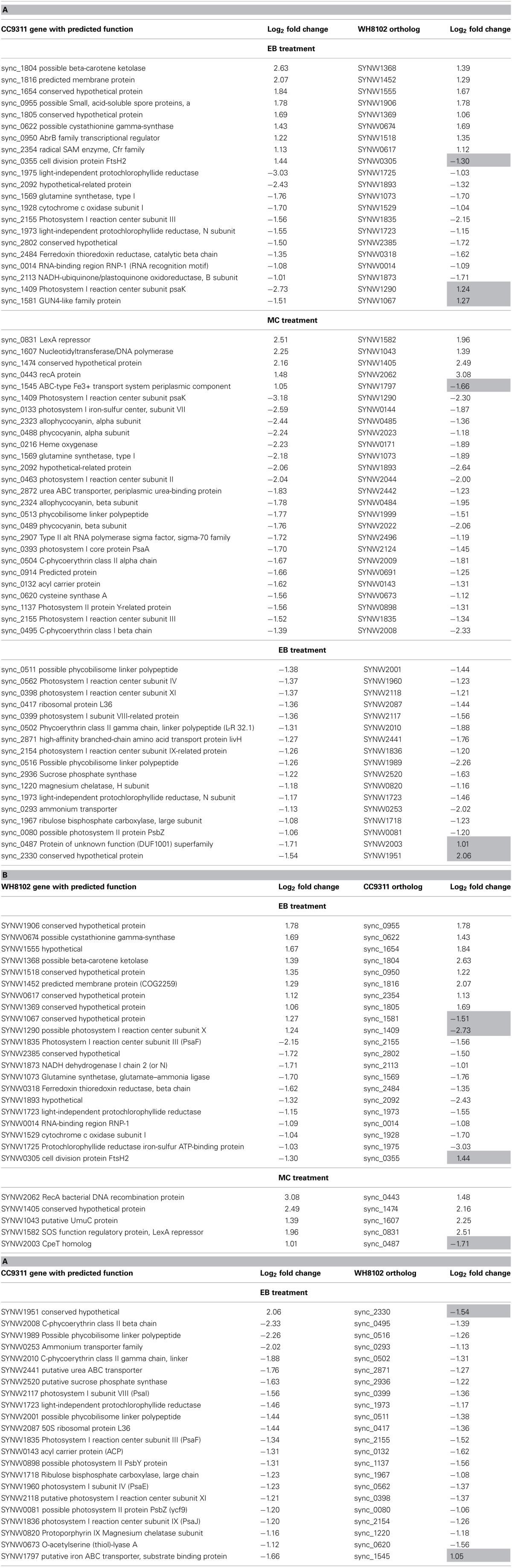
**List of *Synechococcus* sp. CC9311 (A) and WH8102 (B) orthologous genes which showed strong (more than 2-fold) up- or downregulation in both strains in EB or MC treatments**.

*Genes which responded significantly, but in the opposite direction, are highlighted in gray*.

## Supplementary material

Table S1 **Complete list of *Synechococcus* sp. WH8102 and CC9311 genes whose expression was significantly affected by test conditions, according to SAM analysis.** Genes are ordered according to log_2_ fold change (shown in numerator column) with those showing the greatest change in expression under test conditions listed firs.

## References

[B1] Blot N. Mella-Flores D. Six C. Le Corguille G. Boutte C. Peyrat A. (2011). Light history influences the response of the marine cyanobacterium *Synechococcus* sp. WH7803 to oxidative stress. Plant Physiol. 156, 1934–1954 10.1104/pp.111.174714 21670225 PMC3149967

[B2] Bradner W. T. (2001). Mitomycin C: a clinical update. Cancer Treat. Rev. 27, 35–50 10.1053/ctrv.2000.0202 11237776

[B3] Ding X. Z. Bhattacharjee A. Nikolich M. P. Paulsen I. T. Myers G. Seshadri R. (2005). Cloning, expression, and purification of *Brucella suis* outer membrane proteins. Protein Expr. Purif. 40, 134–141 10.1016/j.pep.2004.12.017 15721781

[B4] Domain F. Houot L. Chauvat F. Cassier-Chauvat C. (2004). Function and regulation of the cyanobacterial genes lexA, recA and ruvB: LexA is critical to the survival of cells facing inorganic carbon starvation. Mol. Microbiol. 53, 65–80 10.1111/j.1365-2958.2004.04100.x 15225304

[B5] Dufresne A. Ostrowski M. Scanlan D. J. Garczarek L. Mazard S. Palenik B. P. (2008). Unraveling the genomic mosaic of a ubiquitous genus of marine cyanobacteria. Genome Biol. 9:R90 10.1186/gb-2008-9-5-r90 18507822 PMC2441476

[B6] Dupont C. L. Johnson D. A. Phillippy K. Paulsen I. T. Brahamsha B. Palenik B. (2012). Genetic identification of a high-affinity Ni transporter and the transcriptional response to Ni deprivation in Synechococcus sp. strain WH8102. Appl. Environ. Microbiol. 78, 7822–7832 10.1128/AEM.01739-12 22904052 PMC3485950

[B7] Hassan K. A. Brzoska A. J. Wilson N. L. Eijkelkamp B. A. Brown M. H. Paulsen I. T. (2011). Roles of DHA2 family transporters in drug resistance and iron homeostasis in *Acinetobacter* spp. J. Mol. Microbiol. Biotechnol. 20, 116–124 10.1159/000325367 21430390

[B8] Janion C. (2008). Inducible SOS response system of DNA repair and mutagenesis in *Escherichia coli*. Int. J. Biol. Sci. 4, 338–344 10.7150/ijbs.4.338 18825275 PMC2556049

[B9] Kolowrat C. Partensky F. Mella-Flores D. Le Corguille G. Boutte C. Blot N. (2010). Ultraviolet stress delays chromosome replication in light/dark synchronized cells of the marine cyanobacterium *Prochlorococcus marinus* PCC9511. BMC Microbiol. 10:204 10.1186/1471-2180-10-204 20670397 PMC2921402

[B10] Li S. Xu M. L. Su Z. C. (2010). Computational analysis of LexA regulons in Cyanobacteria. BMC Genomics 11:527 10.1186/1471-2164-11-527 20920248 PMC3091678

[B11] Lindell D. Post A. F. (2001). Ecological aspects of ntcA gene expression and its use as an indicator of the nitrogen status of marine *Synechococcus* spp. Appl. Environ. Microbiol. 67, 3340–3349 10.1128/AEM.67.8.3340-3349.2001 11472902 PMC93026

[B12] Llabres M. Agusti S. (2006). Picophytoplankton cell death induced by UV radiation: evidence for oceanic Atlantic communities. Limnol. Oceanogr. 51, 21–29 10.4319/lo.2006.51.1.0021

[B13] Long R. A. Qureshi A. Faulkner D. J. Azam F. (2003). 2-n-Pentyl-4-quinolinol produced by a marine *Alteromonas* sp. and its potential ecological and biogeochemical roles. Appl. Environ. Microbiol. 69, 568–576 10.1128/AEM.69.1.568-576.2003 12514043 PMC152395

[B14] Mella-Flores D. Six C. Ratin M. Partensky F. Boutte C. Le Corguille G. (2012). *Prochlorococcus* and *Synechococcus* have evolved different adaptive mechanisms to cope with light and UV stress. Front. Microbiol. 3:285 10.3389/fmicb.2012.00285 23024637 PMC3441193

[B15] Mitchell B. A. Paulsen I. T. Brown M. H. Skurray R. A. (1999). Bioenergetics of the staphylococcal multidrug export protein QacA. Identification of distinct binding sites for monovalent and divalent cations. J. Biol. Chem. 274, 3541–3548 10.1074/jbc.274.6.3541 9920900

[B16] Nikaido H. Takatsuka Y. (2009). Mechanisms of RND multidrug efflux pumps. Biochim. Biophys. Acta 1794, 769–781 10.1016/j.bbapap.2008.10.004 19026770 PMC2696896

[B17] Nogales B. Lanfranconi M. P. Pina-Villalonga J. M. Bosch R. (2011). Anthropogenic perturbations in marine microbial communities. FEMS Microbiol. Rev. 35, 275–298 10.1111/j.1574-6976.2010.00248.x 20738403

[B18] Ostrowski M. Mazard S. Tetu S. G. Phillippy K. Johnson A. Palenik B. (2010). PtrA is required for coordinate regulation of gene expression during phosphate stress in a marine *Synechococcus*. ISME J. 4, 908–921 10.1038/ismej.2010.24 20376102

[B19] Palenik B. Brahamsha B. Larimer F. W. Land M. Hauser L. Chain P. (2003). The genome of a motile marine *Synechococcus*. Nature 424, 1037–1042 10.1038/nature01943 12917641

[B20] Palenik B. Ren Q. Dupont C. L. Myers G. S. Heidelberg J. F. Badger J. H. (2006). Genome sequence of *Synechococcus* CC9311: insights into adaptation to a coastal environment. Proc. Natl. Acad. Sci. U.S.A. 103, 13555–13559 10.1073/pnas.0602963103 16938853 PMC1569201

[B21] Partensky F. Hess W. R. Vaulot D. (1999). *Prochlorococcus*, a marine photosynthetic prokaryote of global significance. Microbiol. Mol. Biol. Rev. 63, 106–127 10066832 10.1128/mmbr.63.1.106-127.1999 PMC98958

[B22] Patel D. Kosmidis C. Seo S. M. Kaatz G. W. (2010). Ethidium bromide MIC screening for enhanced efflux pump gene expression or efflux activity in *Staphylococcus aureus*. Antimicrob. Agents Chemother. 54, 5070–5073 10.1128/AAC.01058-10 20855743 PMC2981236

[B23] Patterson-Fortin L. M. Colvin K. R. Owttrim G. W. (2006). A LexA-related protein regulates redox-sensitive expression of the cyanobacterial RNA helicase, crhR. Nucleic Acids Res. 34, 3446–3454 10.1093/nar/gkl426 16840531 PMC1524924

[B24] Peterson S. N. Sung C. K. Cline R. Desai B. V. Snesrud E. C. Luo P. (2004). Identification of competence pheromone responsive genes in *Streptococcus pneumoniae* by use of DNA microarrays. Mol. Microbiol. 51, 1051–1070 10.1046/j.1365-2958.2003.03907.x 14763980

[B25] Richardson J. P. (1973). Mechanism of ethidium bromide inhibition of RNA polymerase. J. Mol. Biol. 78, 703–714 10.1016/0022-2836(73)90290-8 4587136

[B26] Roy Chowdhury A. Bakshi R. Wang J. Yildirir G. Liu B. Pappas-Brown V. (2010). The killing of African trypanosomes by ethidium bromide. PLoS Pathog. 6:e1001226 10.1371/journal.ppat.1001226 21187912 PMC3002999

[B27] Saeed A. I. Sharov V. White J. Li J. Liang W. Bhagabati N. (2003). TM4: a free, open-source system for microarray data management and analysis. Biotechniques 34, 374–378 12613259 10.2144/03342mt01

[B28] Scanlan D. J. Ostrowski M. Mazard S. Dufresne A. Garczarek L. Hess W. R. (2009). Ecological genomics of marine picocyanobacteria. Microbiol. Mol. Biol. Rev. 73, 249–299 10.1128/MMBR.00035-08 19487728 PMC2698417

[B29] Sode K. Oozeki M. Asakawa K. Burgess J. Matusunaga T. (1994). Isolation of a marine cyanophage infecting the unicellular cyanobacterium *Synechococcus* species NKBG 042902. J. Mar. Biotechnol. 1, 189–192

[B30] Soukas A. Cohen P. Socci N. D. Friedman J. M. (2000). Leptin-specific patterns of gene expression in white adipose tissue. Genes Dev. 14, 963–980 10783168 PMC316534

[B31] Stothard P. Wishart D. S. (2005). Circular genome visualization and exploration using CGView. Bioinformatics 21, 537–539 10.1093/bioinformatics/bti054 15479716

[B32] Strom S. L. Brahamsha B. Fredrickson K. A. Apple J. K. Rodriguez A. G. (2012). A giant cell surface protein in *Synechococcus* WH8102 inhibits feeding by a dinoflagellate predator. Environ. Microbiol. 14, 807–816 10.1111/j.1462-2920.2011.02640.x 22103339

[B33] Stuart R. K. Brahamsha B. Busby K. Palenik B. (2013). Genomic island genes in a coastal marine *Synechococcus* strain confer enhanced tolerance to copper and oxidative stress. ISME J. 75, 5047–5057 23344240 10.1038/ismej.2012.175 PMC3660668

[B34] Stuart R. K. Dupont C. L. Johnson D. A. Paulsen I. T. Palenik B. (2009). Coastal strains of marine *Synechococcus* species exhibit increased tolerance to copper shock and a distinctive transcriptional response relative to those of open-ocean strains. Appl. Environ. Microbiol. 75, 5047–5057 10.1128/AEM.00271-09 19502430 PMC2725496

[B35] Su Z. Mao F. Dam P. Wu H. Olman V. Paulsen I. T. (2006). Computational inference experimental validation of the nitrogen assimilation regulatory network in cyanobacterium *Synechococcus* sp. WH (8102). Nucleic Acids Res. 34, 1050–1065 10.1093/nar/gkj496 16473855 PMC1363776

[B36] Tetu S. G. Brahamsha B. Johnson D. A. Tai V. Phillippy K. Palenik B. (2009). Microarray analysis of phosphate regulation in the marine cyanobacterium *Synechococcus* sp. WH8102. ISME J. 3, 835–849 10.1038/ismej.2009.31 19340084

[B37] Thompson A. W. Huang K. Saito M. A. Chisholm S. W. (2011). Transcriptome response of high- and low-light-adapted *Prochlorococcus* strains to changing iron availability. ISME J. 5, 1580–1594 10.1038/ismej.2011.49 21562599 PMC3176520

[B38] Tusher V. G. Tibshirani R. Chu G. (2001). Significance analysis of microarrays applied to the ionizing radiation response. Proc. Natl. Acad. Sci. U.S.A. 98, 5116–5121 10.1073/pnas.091062498 11309499 PMC33173

[B39] Waterbury J. B. Willey J. M. (1988). Isolation and growth of marine planktonic cyanobacteria. Meth. Enzymol. 167, 100–105 10.1016/0076-6879(88)67009-1

[B40] Weiner J. A. Delorenzo M. E. Fulton M. H. (2004). Relationship between uptake capacity and differential toxicity of the herbicide atrazine in selected microalgal species. Aquat. Toxicol. 68, 121–128 10.1016/j.aquatox.2004.03.004 15145222

[B41] Wiegand I. Hilpert K. Hancock R. E. (2008). Agar and broth dilution methods to determine the minimal inhibitory concentration (MIC) of antimicrobial substances. Nat. Protoc. 3, 163–175 10.1038/nprot.2007.521 18274517

